# Supplementary material for: Detection of ESR1 Mutations in Tissue and Liquid Biopsy with Novel Next-Generation Sequencing and Digital Droplet PCR Assays: Insights from Multi-Center Real Life Data of Almost 6000 Patients
Source: Cancers (Basel). 2025 Apr 9;17(8):1266. doi: 10.3390/cancers17081266 (PMC12025842; doi:10.3390/cancers17081266)
Supplement: Supplementary file 1 [file cancers-17-01266-s001.zip › cancers-3540268-supplementary.pdf]

Supplementary Material

Table S1. Genes included in the MH Custom Panel V1 panel used for analysis of pan-cancer cohort of Institute of Pathology, Charité-Universitätsmedizin Berlin, Germany. The panel 2.918 Mbp hybrid-capture SureSelect DNA panel (Agilent Technologies,) designed in 2015 covers whole exonic regions of 624 genes by 111,859 probes.

MH Custom Panel V1				
ABCB1	*ABCC1*	*ABCC2*	*ABCC6*	*ABCG2*
ABL1	*ABL2*	*ACE*	*ACPP*	*ACVRL1*
ADA	*ADAM15*	*AFP*	*AKT1*	*AKT2*
AKT3	*ALK*	*ALOX12*	*ALOX12B*	*ANGPT1*
ANGPT2	*APC*	*AR*	*ARAF*	*ARFRP1*
ARID1A	*ARID2*	*ASXL1*	*ATM*	*ATP1A3*
ATP7A	*ATR*	*ATRX*	*AURKA*	*AURKB*
AURKC	*AVPR2*	*AXL*	*BAD*	*BAP1*
BARD1	*BBC3*	*BCL2*	*BCL2A1*	*BCL2L1*
BCL2L2	*BCL2L3*	*BCL2L4*	*BCL2L5*	*BCL2L6*
BCL2L7	*BCL2L8*	*BCL2L9*	*BCL2L10*	*BCL2L11*
BCL2L12	*BCL2L13*	*BCL2L14*	*BCL2L15*	*BCL2L16*
BRIP1	*BTK*	*BUB1*	*CA9*	*CALCA*
CALCR	*CALM1*	*CALM2*	*CALM3*	*CARD11*
CASP3	*CASP7*	*CASP8*	*CASP9*	*CBFB*
CBL	*CBR3*	*CCL3*	*CCND1*	*CCND2*
CCND3	*CCNE1*	*CCNG1*	*CCR4*	*CCR5*
CD109	*CD151*	*CD19*	*CD22*	*CD248*
CD274	*CD37*	*CD38*	*CD4*	*CD40*
CD44	*CD52*	*CD70*	*CD74*	*CD79A*
CD79B	*CDA*	*CDC7*	*CDC73*	*CDH1*
CDH2	*CDH20*	*CDH5*	*CDK1*	*CDK12*
CDK2	*CDK4*	*CDK6*	*CDK7*	*CDK8*
CDK9	*CDKN1B*	*CDKN2A*	*CDKN2B*	*CDKN2C*
CDKN2D	*CEACAM5*	*CEBPA*	*CENPE*	*CHEK1*
CHEK2	*CIAPIN1*	*CIC*	*CLDN18*	*CLU*
CREBBP	*CRKL*	*CRLF2*	*CSF1R*	*CSF2*
CTCF	*CTLA4*	*CTNNA1*	*CTNNB1*	*CTSG*
CXCL12	*CXCR1*	*CXCR2*	*CYBA*	*CYP11B1*
CYP11B2	*CYP17A1*	*CYP19A1*	*CYP1B1*	*CYP2C19*
CYP2C8	*CYP2C9*	*CYP2D6*	*CYP3A4*	*CYP4B1*
DAXX	*DCT*	*DDR2*	*DDX5*	*DHFR*
DIABLO	*DKK1*	*DLL4*	*DNMT3A*	*DOT1L*
DPP4	*DPYD*	*E2F1*	*EDNRA*	*EDNRB*
EEF2	*EGFL7*	*EGFR*	*EIF4EBP1*	*EML4*
EMSY	*ENG*	*EP300*	*EPCAM*	*EPHA3*
EPHA5	*EPHA6*	*EPHA7*	*EPHB1*	*EPHB4*
EPHB6	*ERBB2*	*ERBB3*	*ERBB4*	*ERCC2*
ERCC5	*ERG*	*ESR1*	*ESR2*	*ETV1*
ETV4	*ETV5*	*ETV6*	*EWSR1*	*EZH2*

MH Custom Panel V1				
F13B	*F2*	*F3*	*F5*	*FAM123B*
FAM46C	*FANCA*	*FANCC*	*FANCD2*	*FANCE*
FANCF	*FANCG*	*FANCL*	*FAS*	*FASLG*
FBXW7	*FGF1*	*FGF10*	*FGF14*	*FGF19*
FGF2	*FGF23*	*FGF3*	*FGF4*	*FGF6*
FGFR1	*FGFR2*	*FGFR3*	*FGFR4*	*FLT1*
FLT3	*FLT3LG*	*FLT4*	*FOLH1*	*FOLR1*
FOXL2	*FOXP4*	*FYN*	*FZD1*	*FZD10*
FZD2	*FZD5*	*FZD7*	*FZD8*	*G6PD*
GATA1	*GATA2*	*GATA3*	*GATA4*	*GDF2*
GGH	*GID4*	*GLP2R*	*GNA11*	*GNA13*
GNAQ	*GNAS*	*GNRHR*	*GPC3*	*GPR124*
GRIN2A	*GRIN3B*	*GRM3*	*GSK3B*	*GSTO1*
GSTO2	*GSTP1*	*GUCY1A2*	*GUSB*	*HBB*
HBEGF	*HDAC1*	*HDAC11*	*HDAC2*	*HDAC6*
HFE	*HGF*	*HIF1A*	*HOXA3*	*HPSE*
HRAS	*HRH2*	*HSP90AA1*	*HSP90AB1*	*HSP90B1*
HSPA5	*IDH1*	*IDH2*	*IFNA2*	*IFNB1*
IFNG	*IGF1R*	*IGF2R*	*IKBKE*	*IKZF1*
IL11RA	*IL13*	*IL13RA2*	*IL1A*	*IL2*
IL21R	*IL25*	*IL29*	*IL2RA*	*IL4*
IL4R	*IL7R*	*INHBA*	*INSR*	*IRF4*
IRS2	*ITGA1*	*ITGA5*	*ITGAM*	*ITGAV*
ITGB1	*ITGB2*	*ITGB3*	*ITGB5*	*ITGB6*
ITK	*ITPA*	*JAK1*	*JAK2*	*JAK3*
JUN	*KDM5A*	*KDM5C*	*KDM6A*	*KDR*
KEAP1	*KIF11*	*KIR2DL1*	*KIT*	*KLF4*
KLHL6	*KLK2*	*KLK3*	*KRAS*	*LAG3*
LGALS1	*LHCGR*	*LOXL2*	*LPA*	*LRP1B*
LRP2	*LRP6*	*LTA*	*LTF*	*LTK*
LYN	*MAGEA1*	*MAGEA4*	*MAP2K1*	*MAP2K2*
MAP2K4	*MAP3K1*	*MAPK1*	*MAPK3*	*MCL1*
MDM2	*MDM4*	*MED1*	*MED12*	*MEF2B*
MEFV	*MEN1*	*MET*	*MGMT*	*MITF*
MLH1	*MLL*	*MLL2*	*MMP2*	*MPL*
MRE11A	*MS4A1*	*MSH2*	*MSH3*	*MSH6*
MST1R	*MSTN*	*MTF1*	*MTHFR*	*MTOR*
MTR	*MUTYH*	*MYC*	*MYCL1*	*MYCN*
MYD88	*MYH11*	*MYST3*	*NAE1*	*NAMPT*
NAT2	*NCF2*	*NCL*	*NF1*	*NF2*
NFE2L2	*NFKB1*	*NFKBIA*	*NGF*	*NKX2-1*
NOD2	*NOTCH1*	*NOTCH2*	*NOTCH3*	*NPM1*
NQO1	*NR4A1*	*NRAS*	*NRP2*	*NTRK1*
NTRK2	*NTRK3*	*NUP93*	*OPRD1*	*P2RX7*
PAK3	*PALB2*	*PARP1*	*PARP2*	*PARP8*
PAX5	*PBRM1*	*PDCD1*	*PDCD1LG2*	*PDGFB*
PDGFRA	*PDGFRB*	*PDK1*	*PDPK1*	*PGF*
PGR	*PHLPP2*	*PIK3CA*	*PIK3CB*	*PIK3CD*

MH Custom Panel V1				
PIK3CG	*PIK3R1*	*PIK3R2*	*PIM1*	*PLA2G10*
PLA2G12A	*PLA2G12B*	*PLA2G1B*	*PLA2G2A*	*PLA2G2D*
PLA2G2E	*PLA2G2F*	*PLA2G3*	*PLA2G5*	*PLA2G6*
PLAU	*PLCG1*	*PLK1*	*PLK4*	*PMP22*
PNP	*POLD1*	*POLE*	*PPARA*	*PPARD*
PPARG	*PPP2R1A*	*PRAME*	*PRDM1*	*PRKAR1A*
PRKCA	*PRKCB*	*PRKDC*	*PRLR*	*PRSS1*
PSMB5	*PSMB8*	*PTCH1*	*PTCH2*	*PTEN*
PTGS2	*PTH*	*PTK2B*	*PTPN11*	*PTPRC*
PTPRD	*RAD50*	*RAD51*	*RAD51L3*	*RAF1*
RARA	*RB1*	*RET*	*RHEB*	*RICTOR*
RNF43	*ROCK2*	*ROS1*	*RPE65*	*RPS27A*
RPTOR	*RRM2*	*RUNX1*	*RUNX3*	*S100A9*
S1PR1	*SELL*	*SELP*	*SERPINA1*	*SERPINE1*
SETD2	*SF3B1*	*SFTPC*	*SHH*	*SLC10A3*
SLC16A1	*SLC19A1*	*SLC29A1*	*SLC5A5*	*SLC6A2*
SLC7A11	*SLCO1B1*	*SMAD2*	*SMAD3*	*SMAD4*
SMARCA4	*SMARCB1*	*SMC4*	*SMO*	*SOCS1*
SOD2	*SOX10*	*SOX2*	*SPEN*	*SPG7*
SPOP	*SPP1*	*SRC*	*SSTR1*	*SSTR2*
SSTR3	*SSTR4*	*SSTR5*	*STAG2*	*STAT3*
STAT4	*STK11*	*SUFU*	*SULT1C4*	*SYK*
T	*TACR1*	*TBX22*	*TEC*	*TEK*
TERT	*TET1*	*TET2*	*TGFB1*	*TGFBR1*
TGFBR2	*TGM2*	*TH*	*TLR2*	*TLR3*
TLR4	*TLR5*	*TLR7*	*TLR8*	*TLR9*
TMPRSS2	*TNC*	*TNF*	*TNFAIP3*	*TNFRSF10A*
TNFRSF10B	*TNFRSF14*	*TNFRSF4*	*TNFRSF8*	*TNFRSF9*
TNFSF10	*TNFSF13*	*TNFSF13B*	*TNKS*	*TOP1*
TOP2A	*TOR1A*	*TP53*	*TP63*	*TP73*
TPMT	*TRPM8*	*TSC1*	*TSC2*	*TSHR*
TUSC2	*TYMS*	*TYR*	*UBA52*	*UBB*
UBC	*UGT1A1*	*UGT1A7*	*UMPS*	*USP9X*
VCAM1	*VDR*	*VEGFB*	*VEGFC*	*VHL*
VKORC1	*VPS4B*	*VWF*	*WISP3*	*WT1*
XIAP	*XPC*	*XPO1*	*XRCC1*	*XRCC2*
YES1	*ZEB2*	*ZNF217*	*ZNF703*	

Table S2. Gene panels used by Hämatopathologie Hamburg (HpH), Germany

HS2-Lung / HS2-Lung-Liquid				
ALK	*CDKN2B*	*IDH1*	*NRG1*	*STK11*
APC	*CTNNB1*	*IDH2*	*NTRK1*	*TP53*
AR	*DDR2*	*KEAP1*	*NTRK2*	
ARID1A	*DPYD*	*KIT*	*NTRK3*	
ATM	*EGFR*	*KRAS*	*PDGFRA*	
ATR	*ERBB2*	*MAP2K1*	*PGR*	
BRAF	*ESR1*	*MAP2K3*	*PIK3CA*	
BRCA1	*FGFR1*	*MDM2*	*POLE*	
BRCA2	*FGFR2*	*MET*	*PTEN*	
CDK4	*FGFR3*	*MTOR*	*RB1*	
CDK6	*FGFR4*	*NFE2L2*	*RET*	
CDKN2A	*HRAS*	*NRAS*	*ROS1*	
NOGGO BRCA und Genome Instability Assay Version 1 (NOGGO GIS V1)				
ABRAXAS1	*BRIP1*	*FANCC*	*MRE11A*	*RAD51B*
APC	*BUB1B*	*FANCD2*	*MSH2*	*RAD51C*
AR	*CDH1*	*FANCE*	*MSH6*	*RAD51D*
ARID1A	*CDK12*	*FANCF*	*NBN*	*RAD52*
ATM	*CHEK1*	*FANCG*	*NRAS*	*RAD54L*
ATR	*CHEK2*	*FANCI*	*PALB2*	*RPA1*
ATRX	*CTNNB1*	*FANCL*	*PIK3CA*	*STK11*
BARD1	*EGFR*	*FANCM*	*PMS2*	*TP53*
BLM	*EMSY*	*HDAC2*	*PPP2A2R*	*XRCC2*
BRAF	*ERBB2*	*HOXB3*	*PTEN*	
BRCA1	*ESR1*	*KRAS*	*RAD50*	
BRCA2	*FANCA*	*MLH1*	*RAD51*	
HS2-Mamma LIQ				
AKT1	*BRCA1*	*ESR1*	*NRAS*	*PGR*
APC	*BRCA2*	*KRAS*	*PIK3CA*	*TP53*
BRAF	*ERBB2*			

Table S3. Details of the SeraSeq ctDNA v4 Mutation Mix 0.5% reference material (SeraCare, USA) used for validation. Chr: chromosome, description: "Gene_Transcript ID_protein change"

chr	start	end	description
chr1	115256529	115256530	NRAS_NM_002524.5_p.Q61R
chr3	178936091	178936092	PIK3CA_NM_006218.4_p.E545K
chr3	178952085	178952086	PIK3CA_NM_006218.4_p.H1047R
chr3	178952149_178952150	178952150	PIK3CA_NM_006218.4_p.N1068Kfs*5
chr6	152419926	152419927	ESR1_NM_000125.4_p.D538G
chr7	140453136	140453137	BRAF_NM_004333.6_p.V600E
chr12	25380275	25380276	KRAS_NM_004985.5_p.Q61H
chr12	25398284	25398285	KRAS_NM_004985.5_p.G12D
chr12	25398285	25398286	KRAS_NM_004985.5_p.G12C
chr13	32936788	32936789	BRCA2_NM_000059.4_p.R2645Nfs*3
chr14	105246551	105246552	AKT1_NM_005163.2_p.E17K
chr17	7577120	7577121	TP53_NM_000546.6_p.R273H
chr17	7577538	7577539	TP53_NM_000546.6_p.R248Q
chr17	7577558	7577559	TP53_NM_000546.6_p.C242Afs*5
chr17	37880984_37880995	37880985	ERBB2_NM_004448.4_p.Y772_A775dup
chr17	41245587	41245588	BRCA1_NM_007294.4_p.K654Sfs*47

Table S4. Details of the Droplex *ESR1* (Transcript ID: NM_000125.4) Mutation Test v2 (Gencurix Inc., Seoul, South Korea)

Exon	Mutant Amino Acid	Oligomix (OM)
Exon 5	p.E380Q	OM2
Exon 7	p.S463P	
Exon 8	p.V534E	OM1
	p.L536R/Q/H/G/K	
	p.Y537S/C/N/H/D	
	p.D538G/E	

Table S5. Reported *ESR1* (Transcript ID: NM_000125.4) amino acid changes in pan-cancer cohort from Hämatopathologie Hamburg (HpH), excluding breast cases (*n* = 5106); LBD: Ligand Binding Domain

Non-LBD					
Protein Change	Frequency	Protein Change	Frequency	Protein Change	Frequency
p.A175D	1	p.G145S	1	p.Q17H	1
p.A175V	1	p.G249E	1	p.R158P	2
p.A223S	1	p.G254V	1	p.R233K	1
p.A288D	1	p.G261*	1	p.R233S	1
p.A50S	1	p.G278V	5	p.R241L	1
p.A569T	3	p.G74S	1	p.R243H	1
p.A58G	1	p.G83V	1	p.R256Q	1
p.A64V	1	p.G83W	1	p.R260S	1
p.A66S	1	p.H216R	1	p.R269C	17
p.A66T	1	p.H267Q	1	p.R269H	3
p.A67V	1	p.H550P	1	p.R269H	3
p.A68P	1	p.H6Y	2	p.R287*	1
p.C227*	1	p.L100Tfs*57	1	p.R548C	1
p.C240*	1	p.L121Cfs*130	1	p.R555H	1
p.C240G	1	p.L14P	5	p.R555S	1
p.C240W	1	p.L165F	1	p.S106G	1
p.D170H	1	p.L35Pfs*51	2	p.S10Y	1
p.D218Y	1	p.L44P	1	p.S118L	1
p.D564G	1	p.M109V	1	p.S137R	1
p.E135*	1	p.M176V	1	p.S554N	1
p.E143Q	1	p.M220I	1	p.S573Y	1
p.E22K	1	p.M220T	1	p.T182N	1
p.E247K	1	p.N101T	1	p.T585M	1
p.E247V	1	p.N153H	1	p.V560M	1
p.E561G	1	p.P107L	1	p.W200L	1
p.E56Rfs*53	1	p.P113L	1	p.Y184C	1
p.E587Rfs*20	1	p.P146Q	1	p.Y60*	1
p.E85K	1	p.P152S	1	p.Y73*	1
p.F149L	1	p.P25T	1		
p.G138del	2	p.P98A	1		
LBD, Non-Hotspot					
Protein Change	Frequency	Protein Change	Frequency	Protein Change	Frequency
p.A312S	1	p.K520R	1	p.P325Q	1

p.C530S	1	p.L319F	1	p.P325Hfs*7	1
p.D313E	1	p.L370F	2	p.P535H	1
p.D321N	1	p.L372F	1	p.Q414H	1
p.D545E	1	p.L408V	1	p.Q500H	1
p.E385D	1	p.L462M	1	p.R434P	2
p.G494D	1	p.L509F	1	p.R503W	1
p.H356D	1	p.N304S	1	p.T311M	1
p.H516N	1	p.N348S	1	p.T334N	1
p.H524Y	1	p.N407D	1	p.V392I	2
p.I326Yfs*17	1	p.P325H	1		
Hotspot					
Protein Change	Frequency	Protein Change	Frequency	Protein Change	Frequency
p.L536H	3	p.S463P	4	p.Y537S	2
p.L536R	2	p.Y537C	1		

Table S6. Reported *ESR1* (NM_000125.4) amino acid changes in pan-cancer cohort from Institute of Pathology, Charité-Universitätsmedizin Berlin (*n* = 863), excluding breast cancer cases**;** LBD: Ligand Binding Domain

Hotspot	
Protein Change	Frequency
p.D538G	1

Table S7. Reported *ESR1* (NM_000125.4) amino acid changes detected after implementation of HS2-Mamma-LIQ assay (n = 354)

Non-LBD					
Protein Change	Frequency	Protein Change	Frequency	Protein Change	Frequency
p.E143K	1	p.K206N	1	p.R183C	2
p.E247K	2	p.M264Tfs*7	2	p.T594M	1
p.G274R	1	p.R157*	1	p.Y246C	1
**LBD, Non-Hotspot**					
Protein Change	Frequency	Protein Change	Frequency	Protein Change	Frequency
p.E380A	1	p.H356D	1	p.N413_G415del	1
p.E380K	1	p.H356Y	2	p.P325Hfs*7	2
p.E542D	1	p.I326Yfs*17	4	p.R363K	1
p.E542K	1	p.L379V	1	p.S329A	1
p.E542Q	1	p.L489V	1	p.S329_E330insL	1
p.F404L	1	p.L536V	1	p.S341L	1
p.F461V	2	p.L536del	1	p.S432L	1
p.G415_C417del	1	p.M357I	1	p.V422del	4
p.G420D	1	p.M421L	1	p.V458L	1
p.G442R	1	p.M543Nfs*12	1		
**Hotspot**					
Protein Change	Frequency	Protein Change	Frequency	Protein Change	Frequency
p.D538G	90	p.L536Q	1	p.Y537D	1
p.E380Q	14	p.L536R	6	p.Y537N	34
p.L536H	4	p.S463P	2	p.Y537S	57
p.L536P	5	p.Y537C	12		

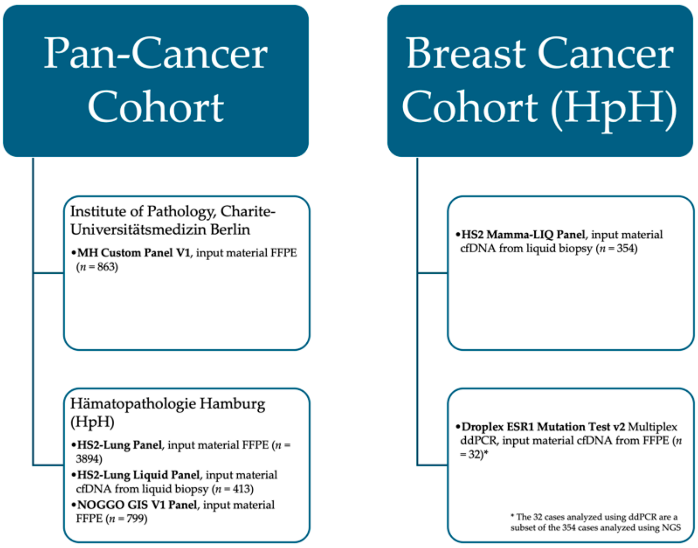

Figure S1. Summary of assays performed for different groups
